# An Active Poroelastic Model for Mechanochemical Patterns in Protoplasmic Droplets of *Physarum polycephalum*

**DOI:** 10.1371/journal.pone.0099220

**Published:** 2014-06-13

**Authors:** Markus Radszuweit, Harald Engel, Markus Bär

**Affiliations:** 1 Weierstraβ-Institut für Angewandte Analysis und Stochastik, Leibniz-Institut im Forschungsverbund Berlin e. V., Berlin, Germany; 2 Institut für Theoretische Physik, Technische Universität Berlin, Berlin, Germany; 3 Physikalisch-Technische Bundesanstalt, Berlin, Germany; Lund University, Sweden

## Abstract

Motivated by recent experimental studies, we derive and analyze a two-dimensional model for the contraction patterns observed in protoplasmic droplets of *Physarum polycephalum*. The model couples a description of an active poroelastic two-phase medium with equations describing the spatiotemporal dynamics of the intracellular free calcium concentration. The poroelastic medium is assumed to consist of an active viscoelastic solid representing the cytoskeleton and a viscous fluid describing the cytosol. The equations for the poroelastic medium are obtained from continuum force balance and include the relevant mechanical fields and an incompressibility condition for the two-phase medium. The reaction-diffusion equations for the calcium dynamics in the protoplasm of Physarum are extended by advective transport due to the flow of the cytosol generated by mechanical stress. Moreover, we assume that the active tension in the solid cytoskeleton is regulated by the calcium concentration in the fluid phase at the same location, which introduces a mechanochemical coupling. A linear stability analysis of the homogeneous state without deformation and cytosolic flows exhibits an oscillatory Turing instability for a large enough mechanochemical coupling strength. Numerical simulations of the model equations reproduce a large variety of wave patterns, including traveling and standing waves, turbulent patterns, rotating spirals and antiphase oscillations in line with experimental observations of contraction patterns in the protoplasmic droplets.

## Introduction

The true slime mold *Physarum polycephalum* is an extensively studied system in biophysics. The plasmodial stage is of particular interest, since it exhibits, despite the relatively simple organization of this unicellular organism, seemingly “intelligent” physiological processes [1]. In this context the term “intelligent” means that, given an external stimulus, the plasmodium optimizes its cell shape, vein network and growth with respect to transport efficiency as well as robustness with respect to link deletion and avoidance of unfavorable conditions [2], [3]. Recent experiments along these lines show that plasmodia were able to reproduce public transport networks on the scale of a petri dish [4] and to “solve” maze problems such as finding the shortest path between two food sources placed at the exits of a labyrinth [5]. Several groups have also investigated the topology and dynamical evolution of the vein network in large Physarum plasmodia with graph theoretical and statistical physics tools [6]–[8]. A second remarkable phenomenon is the synchronization of the contraction patterns in the tubular vein network that generates shuttle streaming to distribute nutrients efficiently throughout the organism [9]. From the perspective of biophysics it is natural to consider these phenomena in the framework of self-organized complex systems [10]. For the formulation of mathematical models a basic understanding of chemical and mechanical processes in the protoplasm is needed.

A first model for strand contraction combined the viscoelastic properties of the ectoplasmic wall with a reaction kinetics that regulates the contractile tension of the actomyosin system [11], [12]. Later, several models in the form of reaction-diffusion [13] and reaction-diffusion-advection equations [14], [15] were formulated that use homogenized quantities, for instance the average strand thickness. These models describe Physarum protoplasm as an oscillatory medium and treat the mechanical feedback in a simplified, qualitative way. More realistic models consider, instead, a two-phase description that distinguishes a fluid sol ( =  cytosol) and a solid gel ( =  cytoskeleton) phase. Some of these models account for sol-gel transformations and were used to explain flow-channel formation [16] and front dynamics [17].

**Figure 1:**
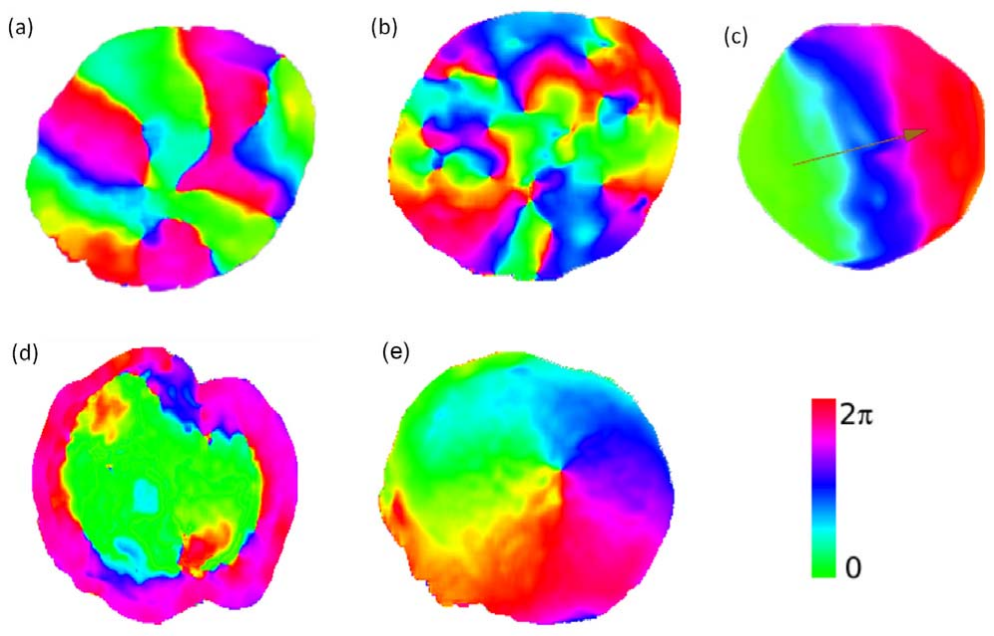
Contractions patterns. Experiments with protoplasmic droplets of Physarum polycephalum [18], [19]. The color represents the local phase of oscillation obtained by a Fourier transformation of the spatiotemporal height data: a) standing wave, b) many irregular spirals, c) traveling wave, d) antiphase patterns, and e) single spiral.

Experiments with microplasmodia, i.e. small plasmodia of sizes ranging from 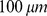 to several millimeters, provide a possibility to study internal amoeboid dynamics of Physarum without the pronounced vein structures usually present in Physarum cells of larger size. Such microplasmodia are produced by extracting cytosol from a Physarum vein and placing it on a substrate. Given a sufficient amount of cytosol, protoplasmic droplets will reorganize and form a new independent cellular entity. During the first hours of this process such cells show a surprising wealth of spatiotemporal mechanical contraction patterns [18], [19] (and U. Strachauer & M.J.B. Hauser, unpublished data). The fact that the cell morphology does not change dramatically and that the cell does not migrate during the first hours, permits observation of the mechanical deformation patterns and waves in a quasi-stationary setting. The observed patterns include spirals, traveling and standing waves as well as antiphase oscillations (see Fig. 1).

Various patterns were reproduced previously by a qualitative particle-based model [20]. However, this description provided no information about the mechanical quantities that are essential to understand the intracellular deformation waves and patterns seen in the experiments.

In a more general context, studying the spatiotemporal instabilities and the related symmetry breaking in intracellular processes has become important to understand many biological processes. In a pioneering paper [21], Turing suggested that the interplay of reactions and diffusion processes provides a fundamental mechanism for morphogenesis. Later, models for intracellular pattern formation that include mechanical forces and the resulting advection processes have been suggested [22], [23]. Active gel models describe the cytoskeleton as an active viscous fluid [24]. In contrast, experiments on inhomogeneous hydration in cells, where large pressure gradients in the cell are observed [25] indicate that the cytoplasm can behave like a porous elastic sponge-like solid (cytoskeleton) penetrated by a viscous fluid phase (cytosol) [26], [27]. Moreover, several multiphase flow models have been proposed as appropriate descriptions of cytoplasmic dynamics [28], [29].

In this paper, we derive and investigate a poroelastic two-phase model of the cytoplasm assuming a viscoelastic solid phase (cytoskeleton) and a fluid phase (cytosol). Furthermore, we incorporate an active tension in the solid phase which is regulated by the concentrations of free calcium ions in the fluid phase, that are in turn advected by the cytosolic flow field. To account for the calcium oscillations observed in experiments with Physarum, a simple one-dimensional active poroelastic model derived earlier [30] is extended to two dimensions and supplemented by a coupling to an oscillatory reaction-diffusion dynamics of the intracellular calcium concentration [31]. Unlike the regulating species in [30], the total free calcium concentration in the model presented here is not conserved anymore and varies strongly in time. As a result, the model displays a short-wavelength instability as opposed to the long-wavelength instability found in [30]. The choice of the poroelastic approach is motivated by the fact that the typical oscillation period of 1 - 2 minutes connected with the spatiotemporal contraction patterns discussed above is considerable shorter than the experimentally observed time of 3 - 6 minutes at which the cytoskeleton starts to exhibit fluid behavior [32]. Hence, the resulting model describes the cytoskeleton as an active viscoelastic solid coupled to a passive fluid in contrast to earlier works that had modeled the cytoskeleton itself as an active fluid [22] addressing long time scales, for which fluidization of the cytoskeleton has already occurred.

The inclusion of the calcium oscillator is necessary because it is known to be essential in the regulation of the contractile actomyosin system of *Physarum polycephalum* [33]. The mechanical and biochemical model parameters are mostly taken from the experimental literature on Physarum and therefore allow quantitative comparisons between model results and experiments. Altogether, in this article we derive and analyze a model for the intracellular dynamics of protoplasmic droplets that treats the cellular mechanics in the framework of a continuous two-phase active poroelastic model coupled to an oscillatory biochemical medium. Active mechanical stresses induce internal pressure gradients that generate cytosolic flow. To account for the latter we extend the equations for the internal calcium oscillator proposed in [31] to a reaction-diffusion-advection (RDA) model closing the mechano-chemical feedback loop. In the methods section we introduce and derive the mathematical mode with a description divided into a mechanical and a biochemical part. Subsequently, the choice of the model parameters is discussed and the numerical methods used to discretize and solve the resulting partial differential equations (PDEs) are described. The next section contains the results obtained by linear stability analysis at the homogeneous steady state (HSS) and a two-parameter phase diagram with numerical simulations. We present also a selection of qualitatively different contraction patterns obtained from simulations of our model, compare them to earlier experimental findings and demonstrate that the variety of patterns found in the experiments with Physarum droplets is reproduced successfully. The paper is concluded with a discussion of the presented model, its limitations and possible extensions.

## Methods

### Model: Mechanical part

**Figure 2:**
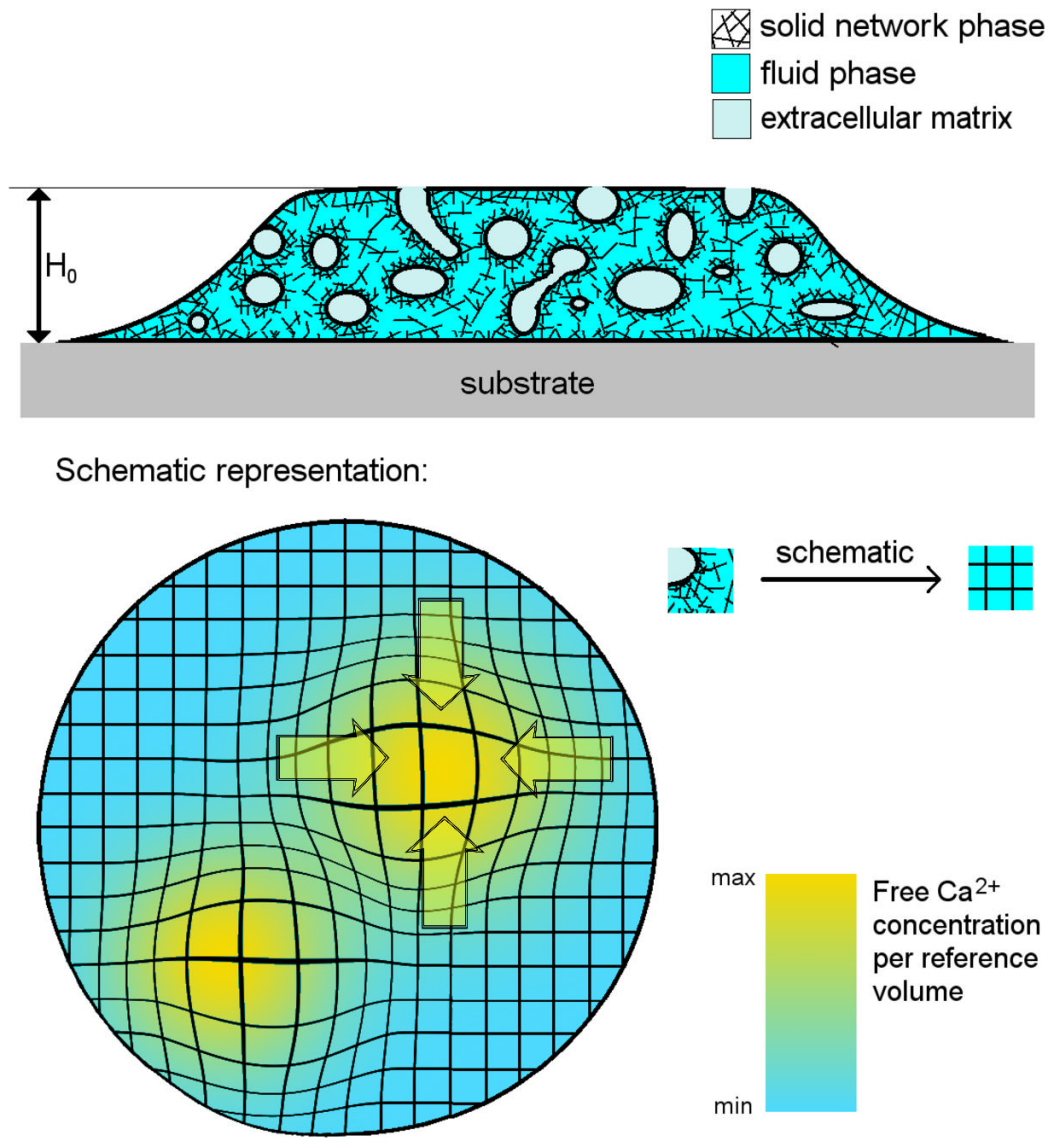
Schematic representation of the the two-phase model. Drawing of a Physarum microdroplet (top) in side view showing the plasmalemma invaginations filled with extracellular matrix (light blue), the fluid phase of the cytoplasm (blue) and the solid filamentous phase (black). Top view of the droplet in the simplified framework of our two-phase model (bottom). Deformations of the cytoskeleton are represented by a distorted grid, flow field in the cytosol by arrows and free 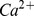 concentration in yellow, respectively.

Physarum protoplasm contains a rudimentary form of an actomyosin system that is also present in cells of higher vertebrates. In contrast to muscle cells the actin filaments in Physarum are randomly oriented in the cortex [34], [35]. In our model, we assume that the cytoplasm contains a solid filamentous phase (gel phase) that has viscoelastic properties and exerts contractile tension on the system. This is illustrated schematically in Fig. 2. The derivation given below is analogous to a recently published generic model for active poroelastic media [30]. Unlike the regulating species in Ref. [30], the total free calcium concentration in the model presented here is not conserved anymore and varies strongly in time. The fluid part of the cytoplasm is modeled as a passive fluid (sol phase) that permeates the cytoskeleton [25]. The velocity field in the sol phase will be expressed by the variable 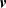. Typical Reynolds numbers that arise from the cytoplasmic flow are small (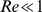 ) and inertia is negligible. The relative volume fraction of solid material is denominated as 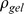 and the fraction of the fluid material as 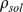 with the additional constraint 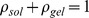.

We define a body-reference coordinate system 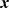 and a displacement field 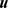 that gives the deviation of the deformed coordinates 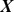: 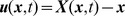 at a time 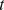. The gel velocity is the substantial time derivative 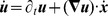 of the displacement field. Since the gel is fixed in the reference coordinate system (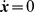), the substantial time derivative 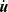 is identical to the partial time derivative 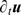.

To determine the flow and displacement field we consider force-balance equations of the form
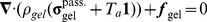
(1)

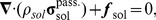
(2)where the passive sol and gel stresses 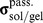 are determined by linear constitutive laws and 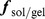 are force densities. The active stress generated in the bulk of the gel phase is expected to be isotropic and hence, given by a multiple of the unit tensor: 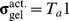. We assume only small deformations and thus restrict ourselves to linear elastic theory. We consider the gel phase as a porous viscoelastic active material [30] that is able to exert contractile stresses by interaction of the myosin-motor system with the actin filaments. The filament orientation in the cortex of Physarum that mainly determines the active and passive properties of the medium is random [35]. Hence, we use an isotropic constitutive law for the elastic stress-strain relation. It has been suggested to consider the cytoplasm as an incompressible medium [36]. In the three-dimensional bulk the total mass flux must be zero. For small strains this can be expressed as [29]:
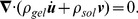
(3)


In the following we assume constant sol and gel fractions 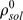 and 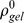 throughout the medium. This is justified, because we consider only small deformations. As a result the transport of cytosol and the related potential inhomogeneities of the fields 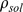 and 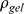 lead only to second-order corrections in the mechanical equations (for details see Text S1 in File S2).

We include a hydrostatic pressure 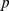 into the stress tensors of sol and gel that originates from the incompressibility of the material expressed by Eq. (3) and neglect the osmotic pressures caused by a difference in the chemical potential (see Text S1 in File S2). We assume a Kelvin-Voigt viscoelastic constitutive law (see e.g. [37]) for the gel phase. Using Darcy's law 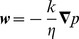 the following relation for the drag force is obtained
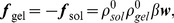
(4)where the parameter 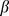 is the ratio between the dynamic viscosity 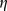 of the cytosol and the permeability 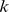 of the porous medium. Instead of the fluid velocity 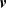 in the laboratory frame, we need to consider the velocity 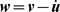 in the body-reference frame introduced above. The sol phase is considered as a passive Newtonian fluid. Hence, only viscous stresses are included for the sol phase. As a result, Eq. (2) corresponds to the Brinkman equation [38]. With the usual expression 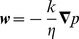 for Darcy's law and Eq. (2) one can relate the coefficient 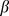 in our model to the viscosity 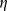 of the cytosol and the permeability 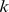 of the medium: 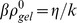. We divide the overall passive stresses in a dissipative and nondissipative (elastic) part:



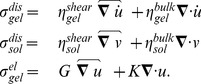
(5)


The factors 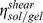 in front of the symmetric traceless parts denote the shear viscosities and 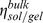 the bulk viscosities for sol and gel phase, respectively. In the elastic stress 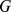 is the shear modulus and 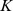 the compression modulus. The mechanical force balance equations in the final form (for details, see Text S1 in File S2) together with the incompressibility condition read:
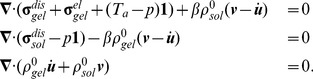
(6)


### Model: Chemical part

Calcium ions play a key role in the regulation of contraction in *Physarum polycephalum*. To describe the calcium kinetics, we use the biochemically realistic model presented by Smith and Saldana [31]. This model describes an oscillation mechanism that is driven by a phosphorylation-dephosphorylation cycle of myosin light chain kinases (MLCKs). A crucial feature of the Smith-Saldana model are autonomous calcium oscillations that occur already in the absence of mechanical feedback. Another entirely different mechanism was introduced to explain oscillations of plasmodial strands of Physarum, where the necessary feedback for the oscillations is provided by mechano-sensitive channels. This feedback acts upon a non-oscillatory calcium reaction kinetics and is therefore required to obtain the oscillations [12], [39]. This model, however, predicts that the free calcium concentration and the mechanical tension oscillate in phase, whereas experiments exhibit an antiphase oscillation with a phase shift of 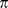 between these two quantities [33]. Moreover, experiments in the homogenate of Physarum plasmodium wherein deformations and mechanical stresses are not possible yield calcium oscillations giving further evidence for an autonomous calcium oscillator [40]. The Smith-Saldana model [31] can be reduced to two ordinary differential equations involving the free calcium concentration 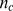 and the fraction of phosphorylated myosin-light-chain kinases 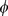:
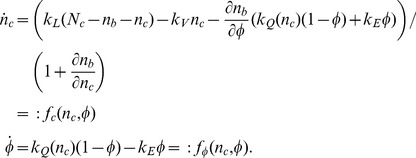
(7)


These equations involve the myosin-bound calcium concentration
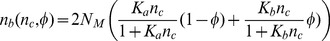
(8)and the calcium-dependent phosphorylation rate of the MLCK
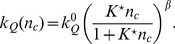
(9)


The chemical parameters introduced in Ref. [31] include the concentration of myosin 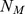, the equilibrium calcium concentration 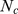, a pumping and leaking rate 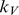 and 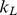 of calcium vacuoles, and the dephosphorylation rate of the MLCK 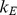. Furthermore, we have the affinity 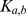 of calcium to the LC1 binding site on the myosin light chain (MLC). The value of this affinity depends on whether the LC2 binding site is phosphorylated (b) or not (a). The AC-cAMP-PKA signal cascade is modeled by Eq. (9) using a Hill-equation with coefficients 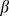, 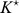, and 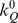. For a detailed explanation of the terms in Eq. (7), we refer the reader to the publications [31], [41]. The model is completed by a third equation that relates the activated fraction of myosin to the active tension 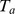. In contrast to the original work of Saldana and Smith, we introduce a relaxation equation analogous to models of cardiac myocytes [42]:
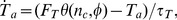
(10)where 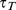 is the relaxation time the tension needs to approach its equilibrium value and 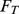 represents the mechanochemical coupling strength. According to the model in Ref. [31] the fraction of activated myosin motors (available for binding to actin) is given by
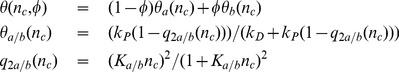
(11)


In this equation two additional parameters, the phosphorylation rate 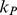 and dephosphorylation rate 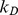 of the LC1 binding site come into play. The relaxation time constant 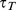 is introduced to obtain a phase shift between calcium concentration and active tension that is 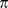. Earlier, a direct proportionality was used [31]. But this work could not reproduce the phase shift between calcium oscillation and active stress that was observed in experiments. Thus, we have adjusted 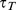 to obtain the correct phase relation.

The next step in the derivation of the model is a spatial extension of Eq. (7) to a reaction-diffusion-advection system. The only species in this model that is transported by diffusion is the free calcium 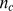. Altogether, the following equation is obtained
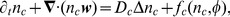
(12)where 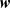 is the fluid velocity in the body-reference frame introduced above and 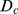 denotes the diffusion constant of the free calcium in the cytosol.

### Summary of the model

Collecting all pieces of the model described so far, we obtain the following two-dimensional system of partial differential equations:
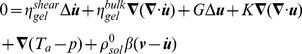
(13)

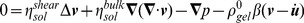
(14)

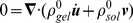
(15)

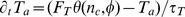
(16)

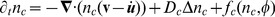
(17)

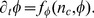
(18)


Here, we have introduced the shear and bulk viscosities 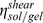 and 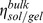 of sol and gel phase and the linear elastic shear and compression modulus of the gel phase 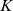 and 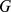, respectively. Note that the sol fraction and the free calcium concentration are given in the body-reference frame. The pressure 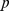 formally appears as a Lagrangian multiplier, due to the incompressibility condition (like in the incompressible Navier-Stokes equation).

The above equations are defined in a circular domain that mimics the geometry of the Physarum droplets in experiments [18], [19] (and U. Strachauer & M.J.B. Hauser, unpublished data). Zero displacement and zero cytosolic flow are imposed at the boundaries. Assuming, a non-permeable membrane of the droplet, no-flux boundary conditions 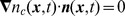 are employed for the calcium concentration 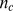, where 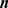 is the normal vector at the boundary. As initial conditions we apply a small random noise 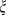 with zero mean as a perturbation to the homogeneous steady state solution.

To compare with the experimentally measured height profiles of the droplet, one needs to estimate the local height field 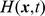 that does not appear explicitly in the two-dimensional model. We relate the relative height deviation 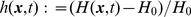
(where 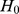 is the height in the reference configuration) to the divergence of the displacement field computed in the two-dimensional model:
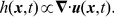
(19)


This approximation is based on the idea that deformations are locally isotropic [35]. Though Eqs. (13)-(18) may appear quite complex at first glance, their essential aspect is the combination of the mechanical dynamics of a poroelastic medium and the calcium oscillator.

### Parameters

A summary of the parameters used in the model is given in Tables 1 and 2. The values for the chemical oscillator are taken from Ref. [31]. For the affinity 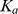 that appears in the functions 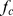 and 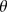 in Eq. (7) two different values are considered. This parameter represents the affinity of the myosin light chain kinase to calcium ions in the unphosphorylated state. Autonomous calcium oscillations are obtained for the parameter 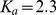 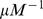, whereas a stationary calcium concentration is found for 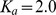 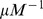 in the absence of mechanical feedback. The effect of the parameter 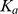 on the autonomous calcium oscillator model for Physarum was studied systematically in Refs. [31], [41]. For the diffusion coefficient of the free calcium we use a typical value of 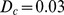 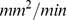 for small ions in cytoplasm [43].

**Table 1:** Mechanical parameters.

Parameter	Value	Description
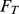	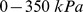	mechanochemical coupling strength [45]
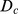	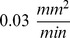	free calcium diffusion coefficient [43]
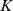	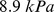	gel compression modulus [45], [46]
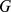	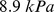	gel shear modulus [45], [46]
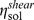	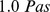	effective sol shear viscosity [49], [50]
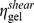	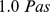	effective gel shear viscosity; indirectly from [51]
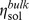	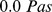	effective sol bulk viscosity
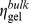	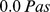	effective gel bulk viscosity
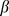	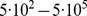 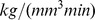	drag coefficient, related to pore size [34], [35]
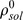	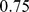	sol volume fraction [44]

Standard parameter set used for the mechanical part of the model given by Eqs. (13) – (15).

**Table 2:** Chemical parameters.

Parameter	Value	Description
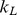	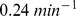	leaking rate of vacuoles [31]
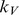	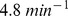	pumping rate of vacuoles [31]
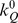	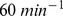	max. phosphorylation rate of MLCK, appears in function 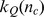 , [31]
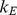	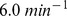	dephosphorylation rate of MLCK [31]
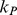	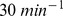	phosphorylation rate of 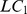
		in function 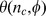 , [31]
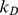	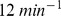	dephosphorylation rate of 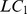
		in function 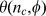 , [31]
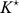	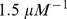	effective activation constant for the
		AC-cAMP-PKA chain, in function 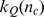 , [31]
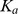	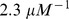	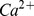 affinity with dephosphorylated MLCK
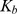	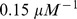	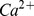 affinity with phosphorylated MLCK [31]
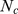	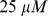	equilibrium total calcium concentration [31]
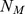	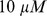	total myosin concentration [31]
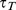	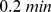	relaxation time for tension generation [41]

Standard parameter set used for the chemical part of the model given by Eqs. (16)-(18).

In Physarum, the percentage of actin in the cytoplasm is estimated to be in the range 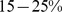 [44]. Since other proteins also contribute to the solid gel phase, we set 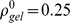 and 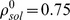. The Young modulus 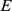 of a Physarum strand was determined to be about 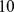 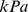 [45]. For sponge-like materials, measurements show that the Poisson ratio is very low: 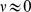 [46] implying 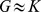 in two dimensions. Therefore, we have set the parameters 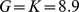 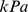.

A typical value for the generated tension in plasmodial strands is 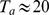 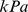 [47]. According to Eq. (16), the active tension 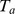 relaxes to an equilibrium value of 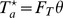. Because 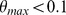
(see Ref. [48]), values up to 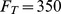 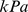 for 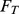 have been considered. Moreover, we have varied 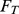 in our study to illustrate the influence of the strength of mechanochemical coupling on the pattern dynamics.

For the dynamic sol viscosity 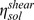 in Physarum, values in the range of 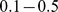 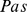 where measured [49]. Alternatively, one can calculate the sol viscosity from velocity profiles measured in Physarum [50]. With this method we obtain a dynamic sol viscosity of around 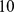 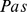. In the model, the value of the sol viscosity is then set to 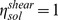 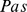. In a study of a composite network containing actin filaments and microtubuli [51] the frequency dependent complex dynamic shear modulus 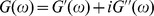 was measured. For a viscoelastic material described by the Kelvin-Voigt model, one identifies 
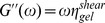
. With a typical frequency 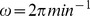 of oscillations in Physarum a value of 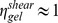 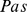 is obtained. For this value of gel viscosity the timescale, on which viscous gel stress is relevant is of the order of 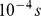. Assuming that the relevant timescale here is about 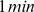, viscous stress in the gel can be neglected. However, due to a stabilizing effect on the numerics, we keep this term. We neglect the influence from the bulk viscosities and set 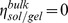. Since the permeability 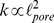, the drag coefficient is 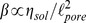, where 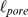 is the average pore size. Porous structures in Physarum exhibit several spatial scales: The actin network pore size is 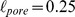 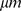 [34] and the membrane structure of invaginations possesses pores with a typical diameter of about 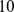 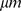 [35]. If one assumes that larger porous structures determine the permeability, a drag coefficient of 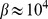 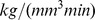 is obtained. Since, however, the porous structure of the Physarum cytoskeleton may vary substantially over time, we have varied the parameter 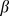 in the range from 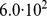 to 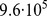 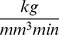.

### Linear stability analysis

Linear stability analysis is used here to investigate the spatiotemporal instability near the homogeneous steady state (HSS) without deformation and without any fluid motion. This HSS is given by 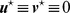, 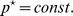 and the steady state values of chemical components and active tension that follow from the implicit equations 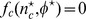, 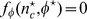 and 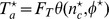. The growth rate and the frequency (for imaginary eigenvalues) of a small perturbation (*δ* 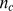, *δ* 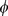, *δ* 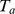, *δ* 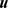, *δ* 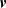, *δ* 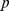)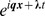 to the HSS is given by the dispersion relation 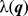.

### Numerical integration

The integration of the full PDE model given by Eqs. (13) – (18) was done with a hybrid method consisting of a finite element (FEM) and finite volume discretization scheme (FVM). This part was implemented as a stand-alone software written in C. Two-dimensional meshes, however, were generated with the free software *Triangle* [52]. With the operator-splitting technique the time integration step was divided into substeps that are carried out with different types of solvers: a fourth order Runge-Kutta method for the nonlinear reaction part, a linear FEM with implicit Euler stepping for the parabolic and elliptic parts and a FVM using the dual mesh of the triangulation (Voronoi diagram) for the advection step. (Operator splitting leads to subproblems of the form 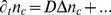
(parabolic PDE), a linear elastic problem 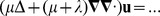
(elliptic PDE) and an advection problem 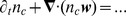 (hyperbolic PDE).) The number of nodes for the mesh discretizing a disc with radius 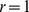 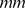 was 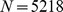. This results in a typical mesh resolution of 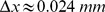, whereas wavelengths obtained in the simulations do not go below 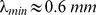. The ratio 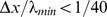 is assumed to provide a sufficient accuracy in order to resolve the profiles of the wave patterns and allow us to qualitatively compare our simulation results with experimental data. The time step size 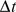 was chosen to be 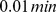 that is about one order of magnitude smaller than the fastest time scale in the reaction kinetics given by Eqs. (16) – (18). The typical simulation length was 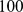 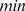.

## Results

### Linear stability analysis and dispersion relation

**Figure 3:**
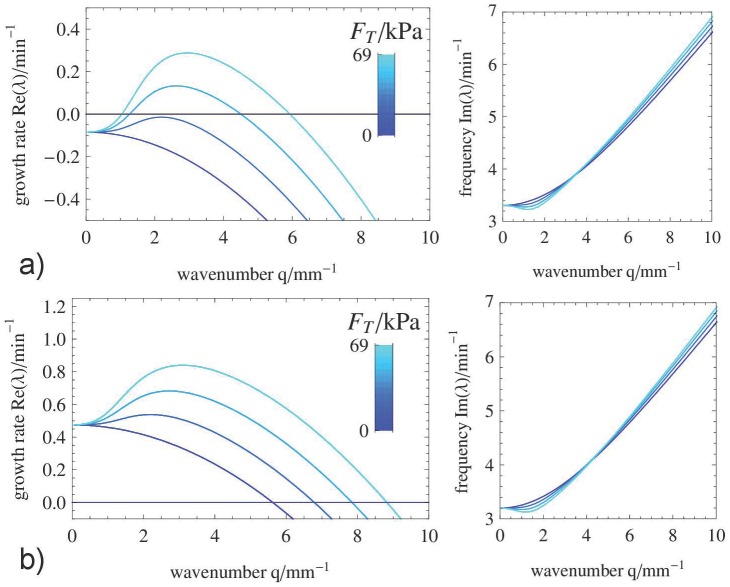
Linear dispersion relation for varying mechanical coupling strength. Real part (left) and imaginary part (right) of the branch of the dispersion relation with largest real parts of the eigenvalues for a) a stable HSS (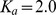 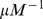) and b) an unstable HSS (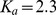 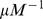). The imaginary part is nonzero for all unstable wavenumbers: 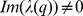. The mechanochemical coupling strength 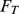 is varied in each case a) and b) increasing from dark to light blue. The drag coefficient is chosen to be 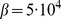 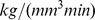 and the remaining parameters are given in Tables 1 and 2.

Here, the mechanochemical coupling strength 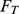 and the drag coefficient 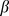 are varied to reveal their influence on the stability of the HSS and the shape of the dispersion curve 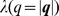 . We have carried out numerical simulations where other model parameters were varied (results not shown) and found that indeed the drag coefficient 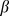 and the coupling strength 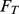 lead to the largest qualitative changes. In contrast, changes in sol viscosity by one order of magnitude had almost no effect on the simulation results. In Fig. 3 the branch of eigenvalues with the largest real part is shown for two scenarios (a and b) with different affinity 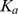 and for different values of 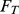. The real and imaginary parts of the dispersion relation are displayed in separate plots. Fig. 3 a) shows the case where the HSS is stable in absence of mechanochemical coupling (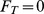). If 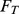 is increased above a critical value, the HSS exhibits a wave instability (oscillatory Turing instability). In Fig. 3 b) we consider a case where the HSS is already unstable against oscillations for 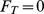, i.e. 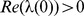. For larger wavenumbers 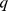 of the perturbation the imaginary part of the growth rate increases indicating that the frequency of waves is larger than the frequency of homogeneous oscillations.

In the case shown in Fig. 3 b), there are two mechanisms that destabilize the HSS: the homogeneous oscillatory mode with 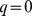 that originates from the calcium kinetics described by Eqs. (7) and a wave-like perturbation at finite wavenumber (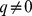) induced by the mechanical feedback. Above a critical mechanochemical coupling strength 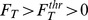 the fastest growing mode (mode with largest real part of the eigenvalue) has a finite wavelength 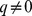 and a nonzero imaginary part indicating wave dynamics.

**Figure 4:**
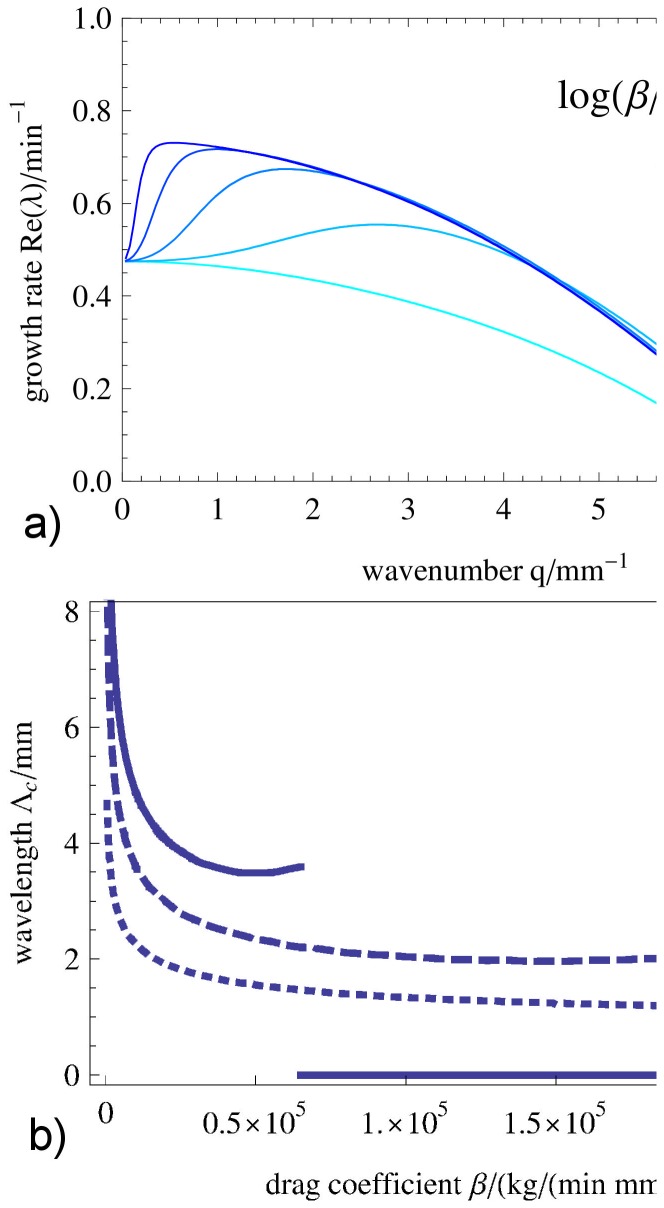
Linear dispersion relation for varying drag coefficient. Branch of the dispersion relation with largest real part a) for different drag coefficients 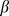 that range over three orders of magnitude (logarithmic scale, from dark blue to cyan). The mechanochemical coupling strength is kept constant at 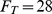 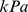. b) Wavelength 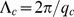 of the fastest growing mode versus drag coefficient 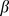 for three different mechanochemical coupling strengths 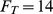 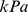 (solid line), 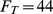 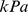 (dashed line) and 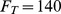 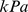 (dotted line). The remaining parameters are given in Tables 1 and 2.

In Fig. 4 a) the influence of variation of the drag coefficient 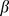 on the dispersion relation for fixed 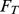 is shown. The wavelength 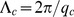 of the perturbation with largest growth rate increases with growing 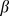 until the maximum with finite wavelength in the dispersion curve disappears. This discontinuity is visible in Fig. 4 b) where 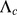 is plotted versus the drag coefficient 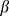 for different values of 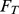. This figure also shows that the fastest growing wavelength 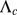 decreases with the mechanochemical coupling strength. Nevertheless, the wavelength depends only weakly on 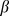 over a large range of values.

The linear stability analysis gives already an important insight into the essential physics of the model defined by Eqs. (13) – (18): The mechanical part leads to a fastest growing mode with non-zero wavenumber while the calcium oscillator can switch the long-wavelength instability observed in the purely mechanical system [30] to a short wavelength instability by removing the conservation constraint for the integrated concentration of free calcium.

### Numerical simulations

**Figure 5:**
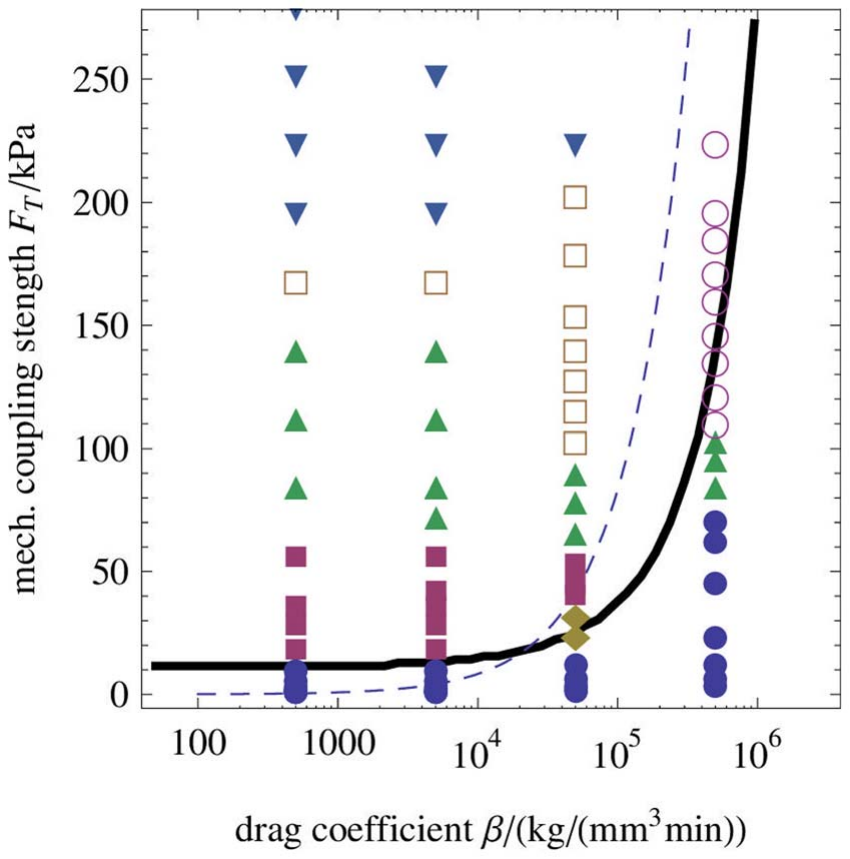
Phase diagram. Phase diagram in the plane spanned by the mechanochemical coupling strength 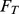 and the drag coefficient 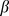. The black line denotes the threshold coupling strength 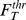, where the most unstable mode according to linear stability analysis of the HSS is nonzero. The dashed blue curve separates the plane according to Eq. 20 in regions with 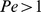
(larger 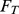) and 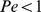
(smaller 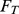).

For different values of the coupling strength 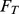 and the drag coefficient 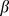, we present numerical simulations of the dynamics of full nonlinear model equations. The corresponding phase diagram is shown in Fig. 5. Therein, the solid black line separates the phase plane according to the shape of the dispersion curve obtained from the linear stability analysis of the HSS: It shows the threshold value 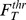 as a function of the parameter 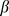. When 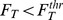 there is no discrete wavenumber 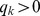, for which 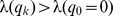. In the opposite case 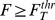 there exists at least one 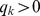 with 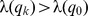. The prediction of homogeneous oscillations for 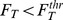 from linear stability analysis is confirmed by numerical simulations for 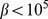 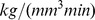 (see Fig. 5, blue discs). However, for larger values of 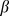 one finds a considerable discrepancy between linear stability analysis and simulation results. For homogeneous oscillations in the calcium concentration the flow and deformation fields both vanish.

To address the question how much influence the advective coupling relative to diffusion has, one can consider a Péclet number that is given by the ratio of diffusive to advective time scales (see also Ref. [22] for a motivation of this definition)
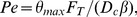
(20) where 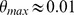 is the typical amplitude for the oscillations in the variable 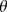 appearing in Eq. (10) for 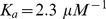. In Fig. 5 the blue line corresponds to 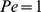. Note, that above the line 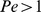, there are no homogeneous oscillations and different types of patterns prevail. In some cases, simple patterns like rotating spirals are traveling waves are also found for 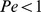. Altogether, this consideration shows that the mechanochemical coupling has to be strong enough to overcome the homogenizing effect of diffusion for mechanochemical waves and patterns to emerge.

**Figure 6:**
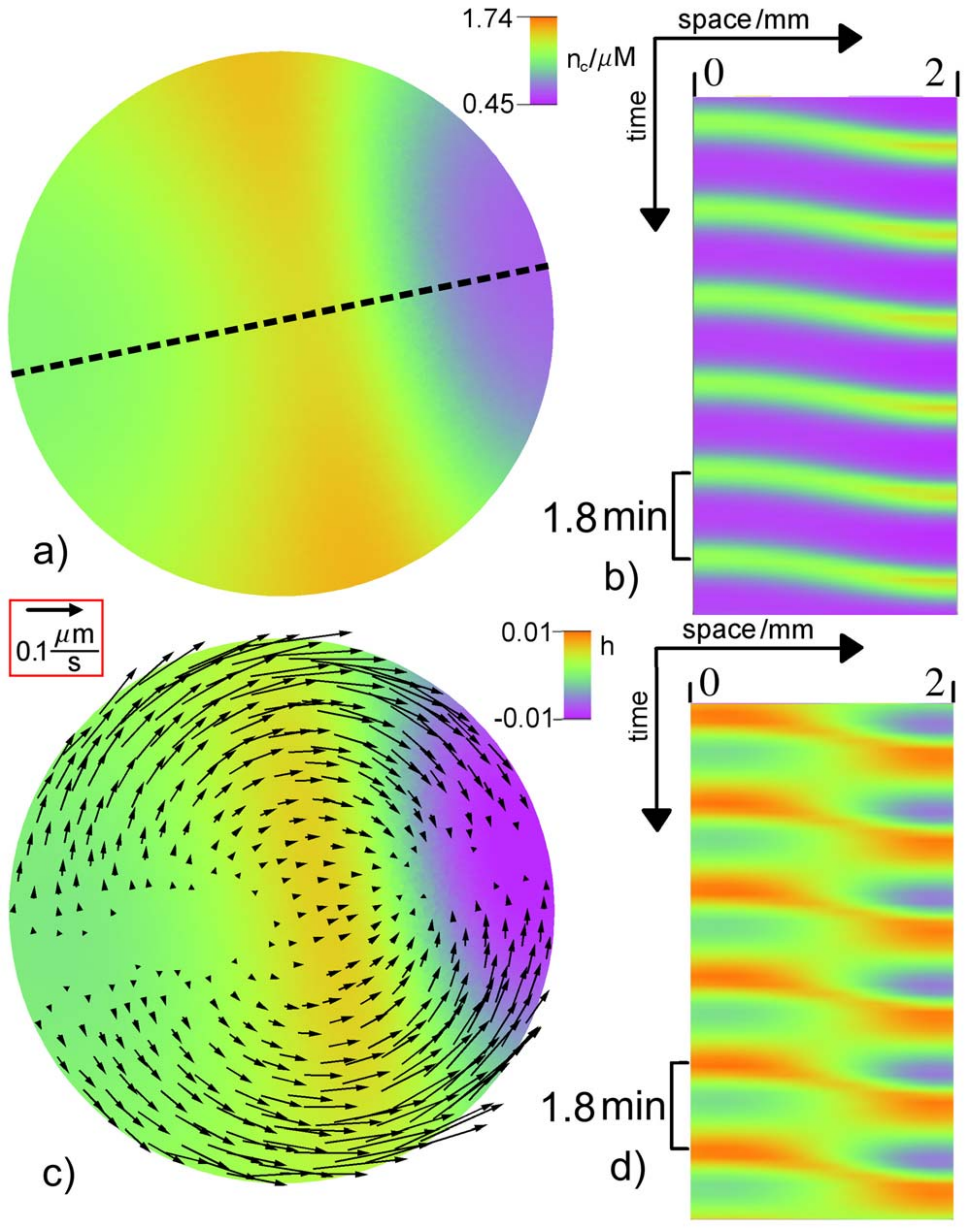
Traveling wave. a) Snapshot of the free calcium concentration 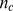 and c) relative height field 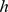 in color and the protoplasmic flow field 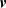 shown by arrows with length 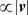. Space-time plot of 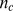 b) and 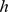 d) along the dotted line in panel a). The period of local oscillations is 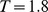 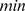. The parameters are 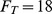 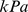 and 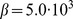 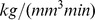. The remaining values can be found in Tables 1 and 2. A video file displaying the spatiotemporal dynamics including the transient initial phase corresponding to subfigure c) is included in the supporting material (see Movie S1 in File S1).

Above 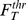, traveling, standing and spiral waves occur in the simulations. In Fig. 6 a traveling wave is depicted by snapshots and space-time plots. In addition to the free calcium concentration 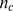 the plots show the relative height field 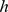 and the sol velocity 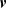, since these quantities are most likely to be measured in experiments. We get an average velocity of the wave front that is about 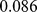 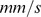.

**Figure 7:**
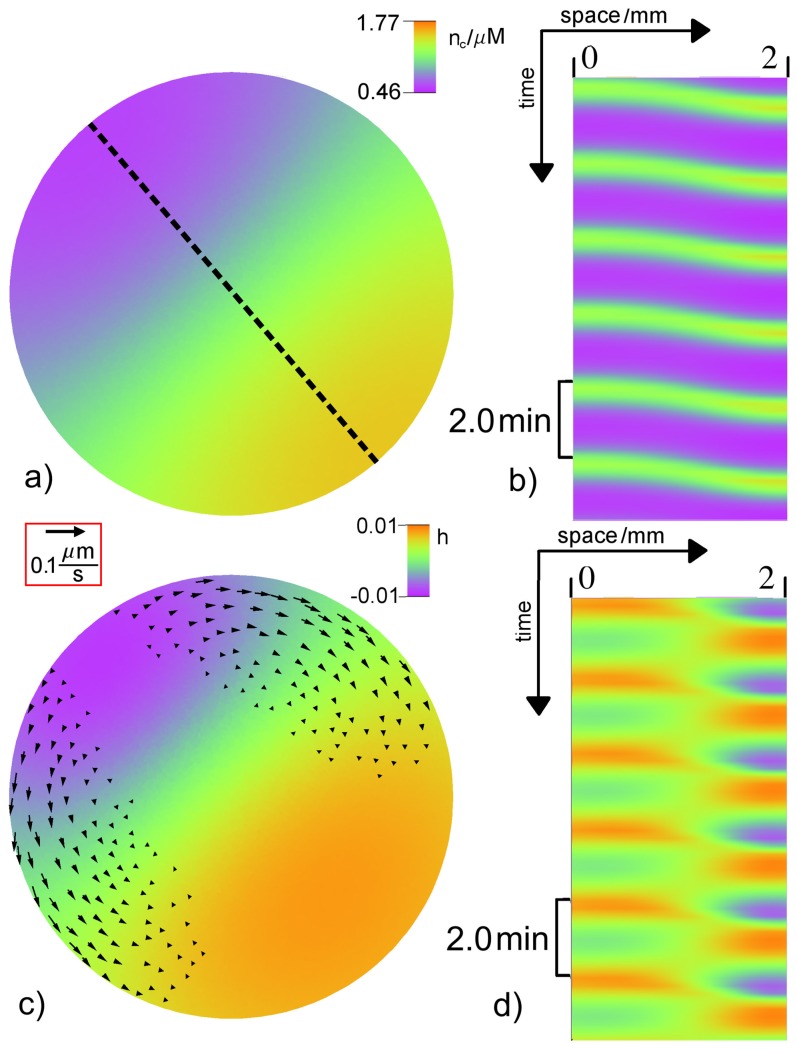
Standing wave. a) Snapshot of the free calcium concentration 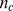 and c) relative height field 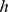 in color and the protoplasmic flow field 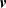 shown by arrows with length 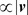. Space-time plot of 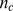 b) and 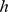 d) along the dotted line in panel a). The period of local oscillations is 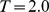 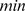. The parameters are 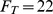 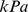 and 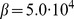 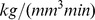. The remaining values can be found in Tables 1 and 2. A video file corresponding to subfigure c) is included in the supporting material (see Movie S2 in File S1).

For values of 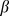 around 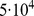 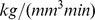 standing waves are observed for the relative height field 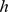, while for the concentration field 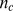 a traveling wave appears (see Fig. 7).

**Figure 8:**
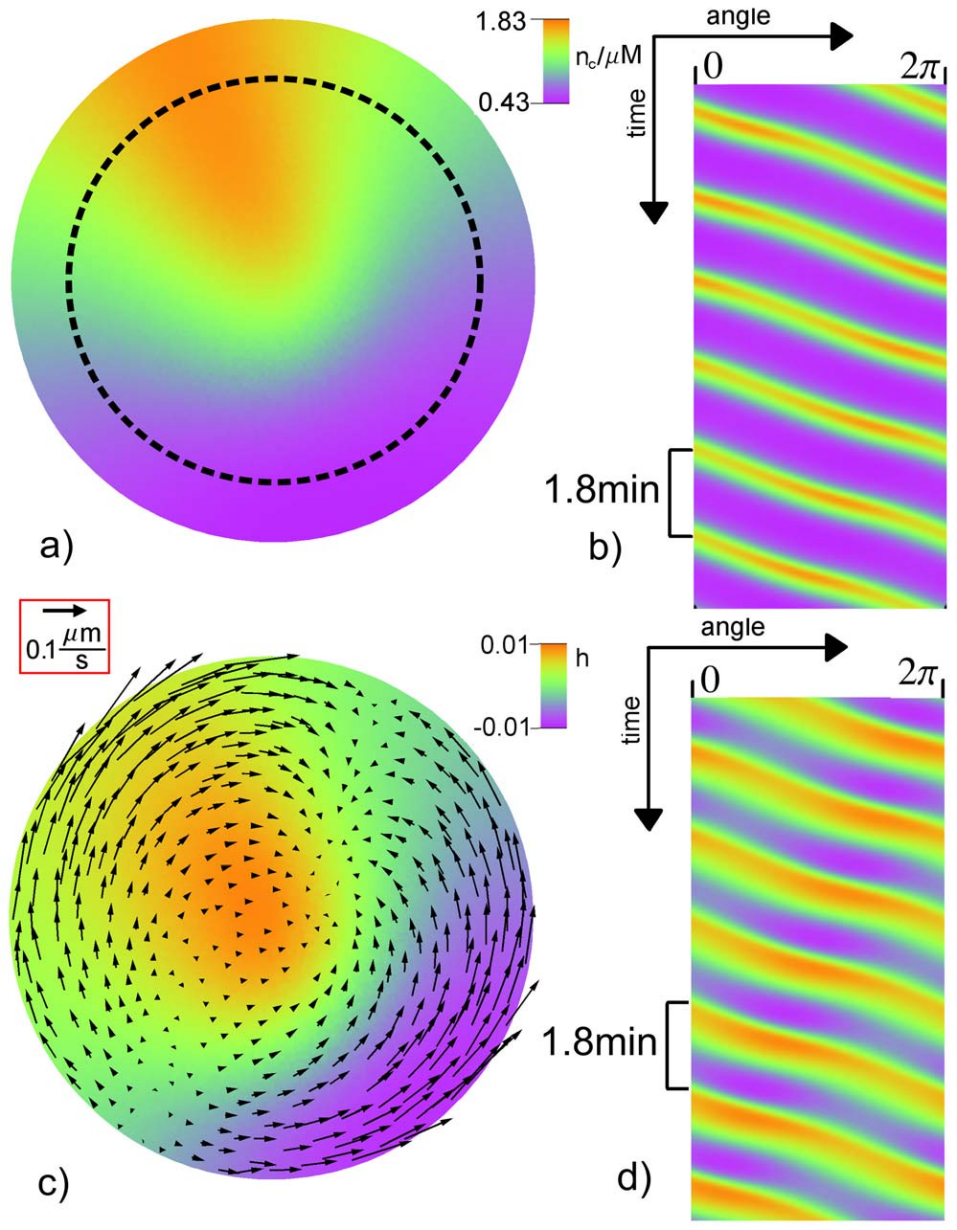
Single rotating spiral. a) Snapshot of the free calcium concentration 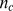 and c) relative height field 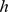 in color and the protoplasmic flow field 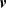 shown by arrows with length 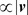. Space-time plot of 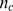 b) and 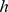 d) along a circle marked by the dotted line in a). The period of local oscillations is 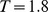 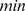. The parameters are 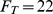 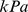 and 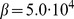 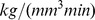. For the remaining values see Tables 1 and 2. A video file corresponding to subfigure c) is included in the supporting material (see Movie S3 in File S1).

Traveling and standing waves often coexist with single rotating spiral patterns. The pattern selection depends on the initial conditions, in particular on the number of phase singularities. Two counter-rotating spirals annihilate and give way to a traveling wave, whereas a single rotating spiral is stable. A single rotating spiral is presented in Fig. 8. No distinct direction of propagation or rotation is preferred reflecting the symmetries of the system. The coexistence region of traveling waves and spirals is marked by violet squares in the phase diagram in Fig. 5. From the space-time plots in Fig. 8 c) one finds a wave speed of 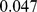 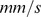.

**Figure 9:**
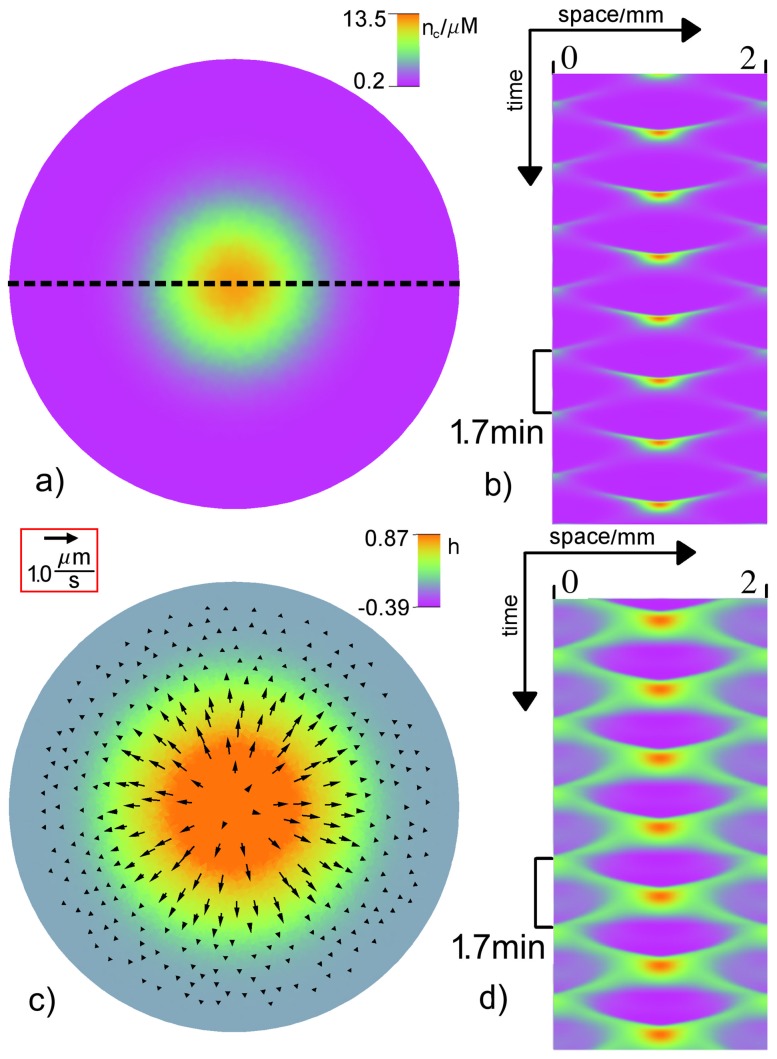
Radial wave. a) Snapshot of the free calcium concentration 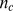 and c) relative height field 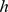 in color and the protoplasmic flow field 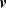 shown by arrows with length 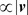. Space-time plot of 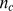 b) and 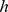 d) along the dotted line in a). The period of local oscillations is 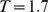 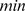. The parameters are 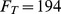 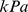 and 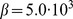 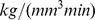. For the remaining values see Tables 1 and 2. A video file corresponding to subfigure c) is included in the supporting material (see Movie S4 in File S1).

For larger mechanochemical coupling strength 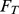, there is no coexistence of spirals with traveling waves: a rotating wave is the only attractor (see Fig. 5, green triangles). For large enough values of the drag coefficient 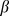 a mode with largest possible wavenumber 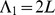 determines the emerging patterns. This is confirmed by the numerical simulations. For 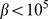 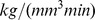 periodic patterns are obtained, even for large coupling 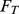 (see Fig. 5, brown squares). These patterns have a characteristic wavelength of the same order as the system size. The simplest pattern is an antiphase oscillations in the form of a radial wave that is reflected at the boundaries (see Fig. 9) that has the symmetry as the experimentally found pattern in Fig. 1d.

**Figure 10:**
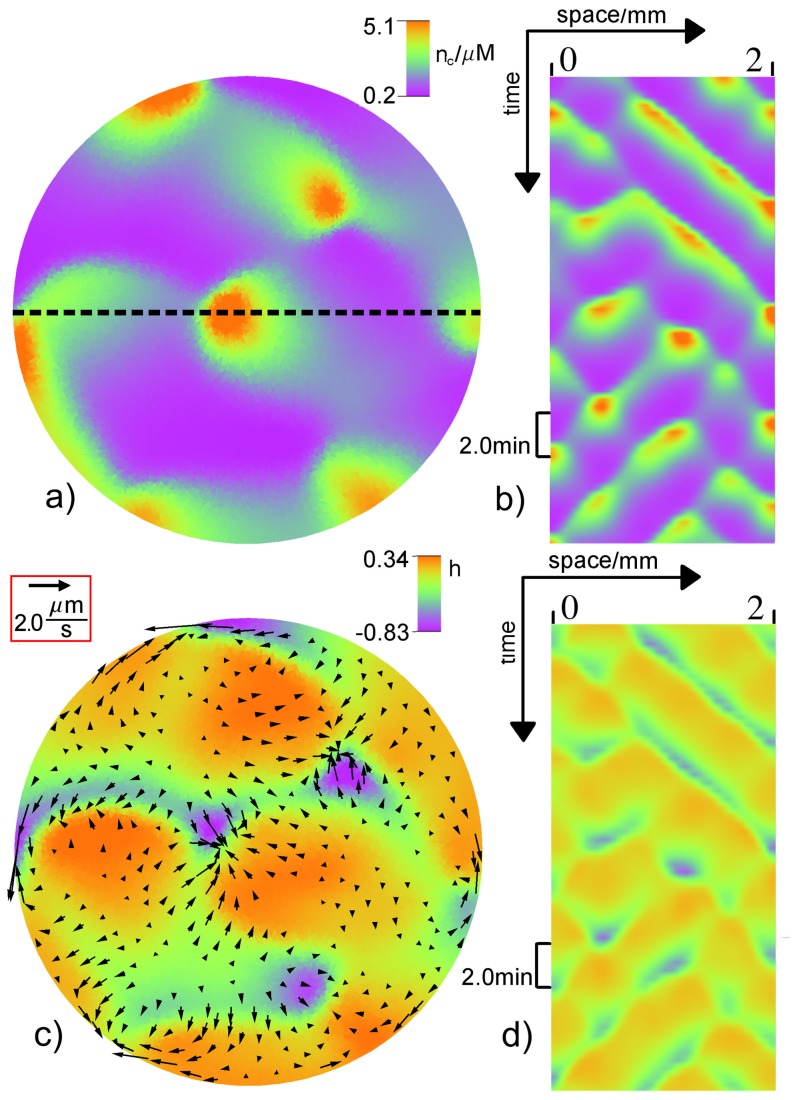
Irregular wave pattern. a) Snapshot of the free calcium concentration 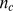 and c) relative height field 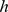 in color and the protoplasmic flow field 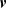 shown by arrows with length 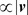. Space-time plot of 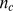 b) and 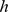 d) along the dotted line in a). The parameters are 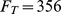 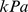 and 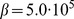 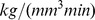. For remaining values see Tables 1 and 2. A video file corresponding to subfigure c) is included in the supporting material (see Movie S5 in File S1).

For large drag coefficients (see Fig. 10 and phase diagram in Fig. 5, pink circles), irregular patterns like the experimental pattern in Fig. 1b with a wavelength significantly smaller than the system size are obtained. For the wave segments in these spatiotemporal patterns we get a typical velocity of 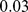 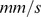 that is much slower than that of traveling or spiral wave.

## Discussion

In this article we have combined a recently published novel mechanical continuum model of the cytoplasm as a poroelastic active medium [30] with the Smith-Saldana model for calcium oscillations in Physarum protoplasm [31]. Upon increase of the mechanochemical coupling strength the homogeneous steady state in this model is destabilized by an oscillatory Turing instability with finite wave number. Homogeneous oscillations of the calcium concentration are replaced by spatiotemporal patterns and waves connected with local deformation and fluid motion. Practically all spatiotemporal contraction patterns observed experimentally in Refs. [18], [19] including rotating spirals, traveling and standing waves, antiphase oscillation and irregular, chaotic waves could be reproduced by numerical simulations of this model. In contrast to earlier models (see e.g. Ref. [20]) for Physarum protoplasm, a closed set of mechanical force-balance equations is derived that allows for explicit computations of pressure and flow fields. The cytoplasm is treated as a two-phase material, consisting of a passive fluid sol phase and an active solid viscoelastic gel phase. The basic idea was already sketched in [41], where however only the case was considered, where the mechanochemical coupling is approximated by a global coupling in the dynamics for calcium. Here, we have instead analyzed and simulated the interplay of mechanical deformation of the cytoskeleton, fluid flow in the cytosol and chemical ( =  calcium) concentration and the associated spatiotemporal dynamics.

Unlike other models that treat the cytoskeleton as a viscoelastic fluid (see e.g. Ref. [53]), we consider the cytoskeleton to be a viscoelastic solid described the Kelvin-Voigt model. This is a valid approximation in Physarum, since the relaxation time of elastic tension is larger by about a factor of three than the calcium oscillation period in the system [32]. The porosity of the solid gel phase allows a flow of cytosol that carries calcium and regulates the tension generation. This is represented by an advective transport of calcium in addition to diffusion and introduces a nonlinear feedback mechanism that couples flows and deformations to a nonlinear reaction kinetics. Related models of active gels and fluids [22], [24], [54] consider the transport of motors, whereas we have considered the transport of calcium that acts as a motor-regulating species in Physarum.

We have limited our considerations to small displacements 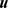 and deformations 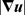. Thus, the equations are formulated up to linear order in the displacement field and its gradient.

A linear stability analysis of a homogeneous steady state with constant calcium concentration and zero deformation and flow has been carried out for the presented model. The resulting dispersion relations reveal that the mechanical feedback provides a new mechanism of an oscillatory Turing instability with nonzero wavenumber if the mechanochemical coupling strength is positive 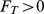. In numerical simulations, homogeneous oscillations get destabilized for sufficiently large coupling strength 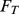. Rotating spirals and traveling waves obtained in the simulations are common patterns found in experiments [18], [19] (and U. Strachauer & M.J.B. Hauser, unpublished data). The wave speed we obtain for traveling and spiral waves agree with the findings in [18]. Note, however, that the antiphase and irregular patterns (Figs. 9 and 10) were obtained at large coupling strength 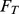 yielding also large deformations. Thus, these results violate the small deformation assumption of our model and should be used only for qualitative comparisons. For a quantitative treatment of large deformations, an extension of the above model to a nonlinear elasticity formulation [55] becomes necessary.

The mechanism of the experimentally observed transition between different patterns on a timescale much larger than the typical calcium oscillation period is not fully understood. The phase diagram in Fig. 5 shows that a minor change in the coupling strength 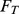 or drag coefficient 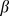 can result in a different type of pattern. Hence, a variation of one of these parameters over the time of the experiment may be responsible for qualitative changes.

For the sake of simplicity, we choose a simple Kelvin-Voigt model as a starting point and found that it is sufficient to reproduce the experimental findings on deformation patterns in Physarum. Future work, e. g. on the modeling of moving droplets of Physarum, may require a more complicated model that exhibits solid behavior of the cytoskeleton at short time scales and fluid behavior at long time scales.

In order to model the stage of migration of a Physarum droplet free-boundary conditions have to be imposed and an interaction with the substrate must be considered (see e.g. [36]). Such an extension of the model may eventually help to understand the interplay of chemical and mechanical processes in the self-organized amoeboid movement of Physarum droplets.

## Supporting Information

Comments about the sol continuity equation, the osmotic pressure, as well as a derivation of the force balance equations from an extremal principle can be found in the Supporting Information (Text S1 in File S2) provided with this article. Furthermore, we include movies (see Movies S1-S5 in File S1) with the spatiotemporal dynamics corresponding to Figs. 6 - 10.

## Supporting Information

File S1
**Supporting movies. Movie S1.** Spatiotemporal dynamics of traveling wave pattern: Relative height field 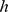 in color and flow field 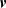 given by arrows corresponding to Fig. 6. The simulation time is 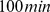 beginning with the initial state. **Movie S2.** Spatiotemporal dynamics of standing wave pattern: Relative height field 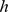 in color and flow field 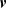 given by arrows corresponding to Fig. 7. The simulation time is 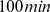 beginning with the initial state. **Movie S3.** Spatiotemporal dynamics of spiral wave pattern: Relative height field 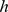 in color and flow field 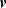 given by arrows corresponding to Fig. 8. The simulation time is 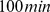 beginning with the initial state. **Movie S4.** Spatiotemporal dynamics of radial wave: Relative height field 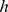 in color and flow field 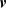 given by arrows corresponding to Fig. 9. The simulation time is 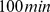 beginning with the initial state. **Movie S5.** Spatiotemporal dynamics of irregular wave pattern: Relative height field 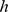 in color and flow field 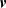 given by arrows corresponding to Fig. 10. The simulation time is 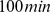 beginning with the initial state.(TAR)

File S2 **Supporting information. Text S1**. This next contains some more detailed information about the derivation of the mechanical model and a comment about the negligence of the osmotic swelling pressure.
(PDF)

## References

[1] Ueda T (2005) An intelligent slime mold: A self-organizing system of cell shape and information. In: Armbruster D, Kaneko K, Mikhailov AS, editors. Networks Of Interacting Machines. Production Organization In Complex Industrial Systems And Biological Cells: World Scientific Publishing. pp. 221–267.

[2] Nakagaki T, Kobayashi R, Nishiura Y, Ueda T (2004) Obtaining multiple separate food sources: behavioural intelligence in the Physarum plasmodium. . Proc. Roy. Soc. B 271: 2305–2310. 10.1098/rspb.2004.2856 PMC1691859 15539357

[3] Nakagaki T, Iima M, Ueda T, Nishiura Y, Saigusa T, et al (2007) Minimum-Risk Path Finding by an Adaptive Amoebal Network. . Phys. Rev. Lett. 99: 068104. 17930872 10.1103/PhysRevLett.99.068104

[4] Tero A, Takagi S, Saigusa T, Ito K, Bebber DP, et al (2010) Rules for biologically inspired adaptive network design. Science 327: 439–442. 20093467 10.1126/science.1177894

[5] Nakagaki T, Yamada H, Tóth Á (2000) Maze-solving by an amoeboid organism. Nature 407: 470. 11028990 10.1038/35035159

[6] Baumgarten W, Ueda T, Hauser MJB (2010) Plasmodial vein network of the slime mold Physarum polycephalum form regular graphs. . Phys. Rev. E 82: 046113. 10.1103/PhysRevE.82.046113 21230351

[7] Fessel A, Oettmeier C, Bernitt E, Gauthier NC, Döbereiner HG (2012) Physarum polycephalum percolation as a paradigm for topological phase transitions in transportation networks. . Phys. Rev. Lett. 102: 078103. 10.1103/PhysRevLett.109.078103 23006405

[8] Baumgarten W, Hauser MJB (2013) Functional organization of the vascular network of Physarum polycephalum. . Phys. Biol. 10: 026003. 23406784 10.1088/1478-3975/10/2/026003

[9] Kamiya N (1981) Physical and chemical basis of cytoplasmic streaming. . Ann. Rev. Plant Physiol. 32: 205–236.

[10] Nakagaki T, Guy RD (2008) Intelligent behaviors of amoeboid movement based on complex dynamics of soft matter. Soft Matter 4: 57–67. 10.1039/b706317m 32907084

[11] Oster GF, Odell GM (1984) Mechanics of cytogels I: Oscillations in Physarum. . Cell Mot. 4: 469–503. 10.1002/cm.970040606 6542453

[12] Teplov VA, Romanovsky YM, Latushkin OA (1991) A continuum model of contraction waves and protoplasm streaming in strands of Physarum plasmodium. Biosystems 24: 269–289. 1863716 10.1016/0303-2647(91)90046-n

[13] Tero A, Kobayashi R, Nakagaki T (2005) A coupled-oscillator model with a conservation law for the rhythmic amoeboid movements of plasmodial slime molds. 2005. Physica D 205: 125–135.

[14] Nakagaki T, Yamada H, Ito M (1998) Reaction-diffusion-advection model for pattern formation of rhythmic contraction in a giant amoeboid cell of the Physarum plasmodium. . J. theor. Biol. 197: 497–506. 10.1006/jtbi.1998.0890 10196092

[15] Yamada H, Nakagaki T, Baker RE, Maini PK (2007) Dispersion relation in oscillatory reaction-diffusion systems with self-consistent flow in true slime mold. . J. Math. Biol. 54: 745–760. 17235581 10.1007/s00285-006-0067-1

[16] Guy RD, Nakagaki T, Wright GB (2011) Flow-induced channel formation in the cytoplasm of motile cells. . Phys. Rev. E 84: 016310. 10.1103/PhysRevE.84.016310 21867307

[17] Ueda K, Takagi S, Nishiura Y, Nakagaki T (2011) Mathematical model for contemplative amoeboid locomotion. . Phys. Rev. E 83: 021916. 10.1103/PhysRevE.83.021916 21405872

[18] Takagi S, Ueda T (2008) Emergence and transitions of dynamic patterns of thickness oscillation of the plasmodium of the true slime mold Physarum polycephalum. Physica D 237: 420–427.

[19] Takagi S, Ueda T (2010) Annihilation and creation of rotating waves by a local light pulse in a protoplasmic droplet of the Physarum plasmodium. Physica D 239: 873–878.

[20] Tsuda S, Jones J (2011) The emergence of synchronization behavior in Physarum polycephalum and its particle approximation. Biosystems 103: 331–341. 21070831 10.1016/j.biosystems.2010.11.001

[21] Turing AM (1952) The chemical basis of morphogenesis. . Phil. Trans. R. Soc. 237: 37–72.

[22] Bois JS, Jülicher F, Grill SW (2011) Pattern formation in active fluids. . Phys. Rev. Lett. 106: 028103. 21405254 10.1103/PhysRevLett.106.028103

[23] Howard J, Grill SW, Bois JS (2011) Turing's next steps: the mechanochemical basis of morphogenesis. . Nat. Rev. Mol. Cell Biol. 12: 392. 21602907 10.1038/nrm3120

[24] Joanny JF, Prost J (2009) Active gels as a description of the actin-myosin cytoskeleton. . HFSP J. 3: 94. 19794818 10.2976/1.3054712 PMC2707794

[25] Charras GT, Mitchison TJ, Mahadevan L (2009) Animal cell hydraulics. . J. Cell Sci. 22: 3233–3241. 10.1242/jcs.049262 PMC2736862 19690051

[26] Moulding DA, Thrasher AJ, Stride E, Mahadevan L, Charras GT (2013) The cytoplasm of living cells behaves as poroelastic material. Nature Materials 12: 253–261. 23291707 10.1038/nmat3517 PMC3925878

[27] Mitchison TJ, Charras GT, Mahadevan L (2008) Implications of a poroelastic cytoplasm for the dynamics of animal cell shape. . Semin. Cell Dev. Biol. 19: 215–223. 18395478 10.1016/j.semcdb.2008.01.008 PMC2696638

[28] Cogan NG, Guy RD (2010) Multiphase flow models of biogels from crawling cells to bacterial biofilms. . HFSP J. 4: 11–25. 20676304 10.2976/1.3291142 PMC2880026

[29] Dembo H, Harlow F (1986) Cell Motion, Contractile Networks, and the Physics of Interpenetrating Reactive Flow. . Biophys. J. 50: 109–121. 3730497 10.1016/S0006-3495(86)83444-0 PMC1329664

[30] Radszuweit M, Alonso S, Engel H, Bär M (2013) Intracellular Mechanochemical Waves in an Active Poroelastic Model. . Phys. Rev. Lett. 110: 138102. 23581377 10.1103/PhysRevLett.110.138102

[31] Smith DA, Saldana R (1992) A model of the *Ca* ^2+^ oscillator for shuttle streaming in Physarum polycephalum. . Biophys. J. 61: 368–380. 1532135 10.1016/S0006-3495(92)81843-X PMC1260253

[32] Nagai R, Yoshimoto Y, Kamiya N (1978) Cyclic production of tension force in the plasmodial strand of Physarum polycephalum and its relation to microfilament morphology. . J. Cell Sci. 33: 205–225. 569161 10.1242/jcs.33.1.205

[33] Yoshimoto Y, Matsumura F, Kamiya N (1981) Simultaneous oscillations of *Ca* ^2+^ efflux and tension generation in the permealized plasmodial strand of Physarum. . Cell Mot. 1: 433–443. 10.1002/cm.970010404 6819083

[34] Nagai R, Kato T (1975) Cytoplasmic Filaments and their Assembly into Bundles in Physarum Plasmodium. Protoplasma 86: 141–158. 1239054 10.1007/BF01275628

[35] Brix K, Kukulies J, Stockem W (1987) Studies on Microplasmodia of Physarum Polycephalum. V. Correlation of Cell Surface Morphology, Microfilament Organization and Motile Activity. Protoplasma 137: 156–167.

[36] Alt W, Dembo M (1999) Cytoplasm dynamics and cell motion: two-phase flow models. . Math. Biosci. 156: 207–228. 10204394 10.1016/s0025-5564(98)10067-6

[37] Banks HT, Hu S, Kenz ZR (2011) A Brief Review of Elasticity and Viscoelasticity for Solids. . Adv. Appl. Math. Mech. 3: 1–51.

[38] Brinkman HC (1949) A calculation of the viscous force excerted by a flowing fluid on a dense swarm of particles. . Appl. Sci. Res. 1: 27–34.

[39] Romanovsky YM, Teplov V (1995) The physical bases of cell movement. The mechanisms of self-organization of amoeboid motility. Phys. Uspekhi 38: 512–543.

[40] Yoshimoto Y, Kamiya N (1982) Ca^2+^ oscillation in the homogenate of Physarum plasmodium. Protoplasma 110: 63–65.

[41] Radszuweit M, Engel H, Bär M (2010) A model for oscillations and pattern formation in protoplasmic droplets of Physarum polycephalum. Eur. Phys. J. Special Topics 191: 159–172.

[42] Panfilov AV, Keldermann RH, Nash MP (2007) Drift and breakup of spiral waves in reaction-diffusion-mechanics systems. Proc. Natl. Acad. Sci. USA 104: 7922–7926. 10.1073/pnas.0701895104 PMC1876548 17468396

[43] Donahue BS, Abercrombie RF (1987) Free diffusion coefficient of ionic calcium in cytoplasm, Cell Calcium. 8: 437–448. 10.1016/0143-4160(87)90027-3 3435913

[44] Kessler D, Nachmias VT, Loewy AG (1976) Actomyosin Content of Physarum Plasmodia and Detection of Immunological Cross-Reactions with Myosins from Related Species. . J. Cell Biol. 69: 393–406. 944188 10.1083/jcb.69.2.393 PMC2109671

[45] Norris CH (1940) Elasticity studies on the myxomycete, *Physarum polycephalum*, . J. Cell. Physiol. 16: 313–322.

[46] Borene ML, Barocas VH, Hubel A (2004) Mechanical and Cellular Changes During Compaction of a Collagen-Sponge-Based Corneal Stromal Equivalent. . Ann. Biomed. Eng. 32: 274–283. 15008375 10.1023/b:abme.0000012747.97620.3a

[47] Wohlfarth-Bottermann K (1977) Oscillating Contractions in Protoplasmic Strands of Physarum: Simultaneous Tensiometry of Longitudinal and Radial Rhythms, Periodicity Analysis and Temperature Dependence. . J. Exp. Biol. 67: 49–59. 561153 10.1242/jeb.67.1.49

[48] Radszuweit M (2013) An Active Poroelastic Model for Cytoplasm and Pattern Formation in Protoplasmic Droplets of Physarum Polycephalum. PhD thesis. Available: http://www2.ub.tu-berlin.de/permalink/eTUB_OPUS3712. 10.1371/journal.pone.0099220 PMC4057197 24927427

[49] Sato M, Wong TZ, Allen RD (1983) Rheological Properties of Living Cytoplasm: Endoplasm of *Physarum* Plasmodium. . J. Cell Biol. 97: 1089–1097. 6619187 10.1083/jcb.97.4.1089 PMC2112616

[50] Bykov AV, Priezzhev AV, Lauri J, Myllylä R (2009) Doppler OCT imaging of cytoplasm shuttle flow in Physarum polycephalum. J. Biophotonics 2: 540–547. 19743444 10.1002/jbio.200910057

[51] Pelletier V, Gal N, Fournier P, Kilfoil ML (2009) Microrheology of Microtubule Solutions and Actin-Microtubule Composite Networks. . Phys. Rev. Lett. 102: 188303. 19518917 10.1103/PhysRevLett.102.188303

[52] Shewchuk JR (1996) Triangle: Engineering a 2D quality mesh generator and Delaunay triangulator. Appl. Comput. Geom 1148: 203–222.

[53] Callan-Jones AC, Jülicher F (2011) Hydrodynamics of active permeating gels. . New. J. Phys. 13: 093027.

[54] Banerjee S, Marchetti MC (2010) Instabilities and oscillations in isotropic active gels. Soft Matter 7: 463–473.

[55] Köpf MH, Pismen LM (2013) Non-equilibrium patterns in polarizable active layers. Physica D 259: 48–54.

