# Supplementary material for: An Active Poroelastic Model for Mechanochemical Patterns in Protoplasmic Droplets of Physarum polycephalum
Source: PLoS One. 2014 Jun 13;9(6):e99220. doi: 10.1371/journal.pone.0099220 (PMC4057197; doi:10.1371/journal.pone.0099220)
Supplement: File S2 — Supporting information. Text S1. This next contains some more detailed information about the derivation of the mechanical model and a comment about the negligence of the osmotic swelling pressure. (PDF) [file pone.0099220.s002.pdf]

An active poroelastic model for mechanochemical patterns in protoplasmic droplets of *Physarum polycephalum*

Supporting Information

Markus Radszuweit, Harald Engel, Markus Bär

Mechanical Equations: A continuity equation for the sol

In the model for Physarum protoplasmic droplets we assume a sponge-like material filled with a fluid that is transported through the pores (see Fig. 2 in the main article). In regions where the influx of sol exceeds the outflux, the sponge will expand. As a result the sol fraction increases. It is a basic assumption in our model that the solid (gel) fraction in the body-reference coordinate frame is constant in space and time: $\rho_{gel} = \rho_{gel}^0 = const.$ Given the deformation gradient $\boldsymbol{F} = \boldsymbol{\nabla}_{\boldsymbol{x}} \boldsymbol{X} = \boldsymbol{1} + \boldsymbol{\nabla} \boldsymbol{u}$, the sol/gel fractions $\varrho_{sol/gel}$ in the lab frame are

$$\begin{aligned} \rho_{sol} &= \varrho_{sol} det\boldsymbol{F} \\ \rho_{gel} &= \varrho_{gel} det\boldsymbol{F}. \end{aligned} \tag{1}$$

The relation $\varrho_{sol} + \varrho_{gel} = 1$ holds exactly in the lab frame, while in the reference frame we have:

$$\rho_{sol} + \rho_{gel} = det\boldsymbol{F} = 1 + \boldsymbol{\nabla} \cdot \boldsymbol{u} + \mathcal{O}(\boldsymbol{\nabla} \boldsymbol{u}^2). \tag{2}$$

Since ρ_{gel} is a constant we arrive at the simple relation (with $\rho_{sol}^0 := 1 - \rho_{gel}^0$)

$$\rho_{sol} = \rho_{sol}^0 + \boldsymbol{\nabla} \cdot \boldsymbol{u} + \mathcal{O}(\boldsymbol{\nabla} \boldsymbol{u}^2). \tag{3}$$

To linear order in $\boldsymbol{\nabla} \boldsymbol{u}$ (small strains) the incompressibility condition reads (see also [1] and [2])

$$\boldsymbol{\nabla} \cdot (\rho_{sol}^0 \boldsymbol{v} + \rho_{gel}^0 \dot{\boldsymbol{u}}) = 0. \tag{4}$$

Using Eqs. (3) and (4) a continuity equation of the form

$$\dot{\rho}_{sol} + \boldsymbol{\nabla} \cdot ((\boldsymbol{v} - \dot{\boldsymbol{u}})\rho_{sol}) = 0 \tag{5}$$

is derived that is valid in linear order in $\boldsymbol{\nabla u}$. With this we show the analogy to other models of two-phase flow [3, 4]. Here, we neglect sol-gel transformations. For the presented mechanical model we only need the zeroth order in ρ since higher order corrections lead to second order terms of $\boldsymbol{\nabla u}$ in the force balance equations (see supplementary material in [1]).

Mechanical Equations: Derivation of the Force-Balance Equations

The sol and gel stresses are divided into a dissipative and non-dissipative part: $\boldsymbol{\sigma}_{gel} = \boldsymbol{\sigma}_{gel}^{non} + \boldsymbol{\sigma}_{gel}^{dis}$ and $\boldsymbol{\sigma}_{sol} = \boldsymbol{\sigma}_{sol}^{non} + \boldsymbol{\sigma}_{sol}^{dis}$. First we consider only the dissipative part, write a functional for the entropy production rate and minimize that functional compare also e.g. [3]. This assumes local thermodynamic equilibrium. We use the summation convention and write the functional:

$$\begin{aligned} -T\dot{S} &= J^{dis}[\dot{\boldsymbol{u}}, \boldsymbol{v}, p] = -\tfrac{1}{2}\int_{\mathcal{B}} d\boldsymbol{x} (\rho^0_{gel}\sigma^{dis}_{gel\alpha\beta}\partial_\beta \dot{u}_\alpha + \rho^0_{sol}\sigma^{dis}_{sol\alpha\beta}\partial_\beta v_\alpha \\ &\quad +\beta\rho^0_{gel}\rho^0_{sol}(\dot{u}_\alpha - v_\alpha)(\dot{u}_\alpha - v_\alpha) - 2p(\rho^0_{gel}\partial_\alpha \dot{u}_\alpha + \rho^0_{sol}\partial_\alpha v_\alpha). \end{aligned} \tag{6}$$

The minimization is carried out under the constraint of incompressibility, leading to an additional hydrostatic pressure field p. The quantities $\rho_{sol/gel}$ are treated as constants (see above). We consider the situation where $|\boldsymbol{\nabla u}| \ll 1$. The resulting Euler-Lagrange equations are:

$$\frac{\partial j^{dis}}{\partial \dot{u}_\gamma} - \partial_\nu (\frac{\partial j^{dis}}{\partial(\partial_\nu \dot{u}_\gamma)}) = 0 \tag{7}$$

$$\frac{\partial j^{dis}}{\partial v_\gamma} - \partial_\nu (\frac{\partial j^{dis}}{\partial(\partial_\nu v_\gamma)}) = 0 \tag{8}$$

$$\frac{\partial j^{dis}}{\partial p} - \partial_\nu (\frac{\partial j^{dis}}{\partial(\partial_\nu p)}) = 0. \tag{9}$$

Linear constitutive laws for an isotropic viscous medium are used that involve the shear and bulk viscosities of sol and gel phase:

$$\begin{aligned} \sigma^{dis}_{gel\alpha\beta} &= \eta^{shear}_{gel}(\partial_\beta \dot{u}_\alpha + \partial_\alpha \dot{u}_\beta - \tfrac{2}{d}\partial_\lambda \dot{u}_\lambda \delta_{\alpha\beta}) + \eta^{bulk}_{gel}\partial_\lambda \dot{u}_\lambda \delta_{\alpha\beta} \\ \sigma^{dis}_{sol\alpha\beta} &= \eta^{shear}_{sol}(\partial_\beta v_\alpha + \partial_\alpha v_\beta - \tfrac{2}{d}\partial_\lambda v_\lambda \delta_{\alpha\beta}) + \eta^{bulk}_{sol}\partial_\lambda v_\lambda \delta_{\alpha\beta} \end{aligned} \tag{10}$$

We keep the spatial dimension d as a variable in the supplementary material. To obtain the equations in the main text one has to set $d = 2$. Eqs. (7) and (8) yield the force balance equations

$$-\beta\rho_{sol}\rho_{gel}(\dot{u}_\gamma - v_\gamma) + \rho_{gel}\partial_\nu(\sigma^{dis}_{gel\gamma\nu} - p\delta_{\gamma\nu}) = 0 \tag{11}$$

$$\beta\rho_{sol}\rho_{gel}(\dot{u}_\gamma - v_\gamma) + \rho_{sol}\partial_\nu(\sigma^{dis}_{sol\gamma\nu} - p\delta_{\gamma\nu}) = 0, \tag{12}$$

while Eq. (9) reproduces the incompressibility condition.

So far, we only included viscous stresses. However, the gel phase should be active and elastic. The Kelvin-Voigt model of viscoelasticity is incorporated by replacing the purely viscous gel stress discussed so far by the sum of viscous elastic and active stress: $\boldsymbol{\sigma}^{dis}_{gel} \to \boldsymbol{\sigma}^{dis}_{gel} + \boldsymbol{\sigma}^{el}_{gel} + \boldsymbol{\sigma}^{act}_{gel}$.

The general linear isotropic constitutive law for an elastic solid is:

$$\sigma^{el}_{gel\alpha\beta} = G(\partial_\beta u_\alpha + \partial_\alpha u_\beta - \frac{2}{d}\partial_\lambda u_\lambda \delta_{\alpha\beta}) + K\partial_\lambda u_\lambda \delta_{\alpha\beta} \tag{13}$$

with shear modulus G and compression modulus K. The active stress generated by the actomyosin system is also considered as isotropic:

$$\sigma^{act}_{gel\alpha\beta} = T_a \delta_{\alpha\beta}. \tag{14}$$

Summing Eqs. (10),(13) and (14) up to the total gel stress and inserting it into Eq. (11) one obtains Eq. (9) in the main article; Eq. (12) corresponds to Eq. (10) in the main article.

Osmotic swelling pressure

It was stipulated in [5] that in a highly concentrated polymer solution like cytoplasm there should by an additional gel pressure $p_{osm} = f(\varrho_{gel})$ that causes a swelling with increasing concentration $p \to p + p_{osm}$. In our model the osmotic stresses due to differences in the concentration of the actin phase are neglected. The following considerations will justify this step. In the framework of the small strain theory we can expand:

$$p_{osm}(\varrho_{gel}) \approx p_{osm}(\rho^0_{gel}) - \frac{\partial f}{\partial \varrho_{gel}}(\rho^0_{gel})\rho^0_{gel}\partial_\alpha u_\alpha. \tag{15}$$

We add this to the hydrostatic pressure in Eq. (11) and get the following terms:

$$...+G\partial_{\alpha\alpha}u_\beta+(K+\frac{d-2}{2}G)\partial_{\alpha\beta}u_\alpha+\frac{\partial f}{\partial\varrho_{gel}}(\rho^0_{gel})\rho^0_{gel}\partial_{\alpha\beta}u_\alpha+\partial_\beta(T_a-p)+... \quad (16)$$

It becomes obvious that one can absorb the osmotic pressure term in the compression modulus:

$$K'=K+\frac{\partial f}{\partial\varrho_{gel}}(\rho^0_{gel})\rho^0_{gel}. \quad (17)$$

Consequently, including this form of osmotic pressure does not lead (in the linear approximation) to any behavior that is not captured by the model presented here.

References

1. Radszuweit M, Alonso S, Engel H, Bär M (2013) Intracellular Mechanochemical Waves in an Active Poroelastic Model. Phys. Rev. Lett. 110: 138102

2. Radszuweit M (2013) An Active Poroelastic Model for Cytoplasm and Pattern Formation in Protoplasmic Droplets of Physarum Polycephalum. PhD thesis. Available: http://www2.ub.tu-berlin.de/permalink/eTUB_OPUS3712

3. Alt W, Dembo M (1999) Cytoplasm dynamics and cell motion: two-phase flow models. Math. Biosci. 156: 207-228

4. Cogan NG, Guy RD (2010) Multiphase flow models of biogels from crawling cells to bacterial biofilms. HFSP J. 4: 11-25

5. Oster GF, Odell GM (1984) Mechanics of cytogels I: Oscillations in Physarum. Cell Mot. 4: 469-503
